# Evolution of the COVID-19 pandemic over six weeks in four French-speaking countries in West Africa

**DOI:** 10.7189/jogh.11.03008

**Published:** 2021-01-16

**Authors:** Tamba Mina Millimouno, Mohamed Ali Ag Ahmed, Birama Apho Ly, Alexandre Delamou, Boukary Sana, Christophe Laba Faye, Wim Van Damme

**Affiliations:** 1 National Training and Research Center in Rural Health of Maferinyah, Forecariah, Guinea; 2 Department of Public Health, Institute of Tropical Medicine, Antwerp, Belgium; 3 Faculty of Medicine and Dentistry, University of Sciences, Techniques and Technologies of Bamako, Bamako, Mali; 4 Faculty of Pharmacy, University of Sciences, Techniques and Technologies of Bamako, Bamako, Mali; 5 Africa Center of Excellence for the Prevention and Control of Transmissible Diseases, Gamal Abdel Nasser University of Conakry, Conakry, Guinea; 6 Management Sciences for Health, Ouagadougou, Burkina Faso; 7 Department of Migration Health, International Organization for Migration, Dakar, Senegal

The novel Coronavirus disease 2019 (COVID-19; caused by the virus SARS-CoV-2) is severely challenging health systems worldwide. In Africa, the first case of COVID-19 was reported by Egypt on February 15, 2020, after which the pandemic spread to all countries on the continent with 46 829 positive cases as of May 12, 2020 [1]. Countries are experiencing different trajectories of disease progression, with obvious differences in the epidemiological surveillance indicators between countries. Here, we describe the evolution of the COVID-19 pandemic in Guinea, Mali, Senegal and Burkina Faso over the first six weeks and discuss the factors that might explain differences in the disease progression.

This study was conducted as part of a collaborative project called “COVID-19 in Francophone Africa”. The countries participating in this project were selected on the basis of a call for applications as part of an initiative by the “Francophone Africa and Fragility” (AFRAFRA) network, which brings together more than 100 national and international experts. We analyzed four surveillance indicators (number of screening tests, number of confirmed cases, positivity rate and case fatality rate) whose data were regularly available from the daily situation reports published online by health authorities in the four countries. Aggregated data were extracted from these reports, as reported by the National Health Security Agency in Guinea [2], the Ministry of Health (MoH) and Social Affairs in Mali [3], the MoH and Social Action in Senegal [4], and the Government of Burkina Faso [5], and entered into a standardized Microsoft Excel spreadsheet which was imported into the STATA software version 15 for analysis (Stata Corp, College Station, TX, USA).

## TRENDS IN COVID-19 INCIDENCE

**Figure 1:**
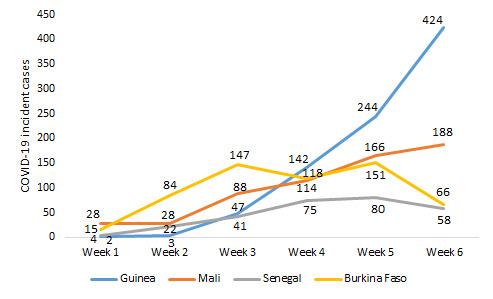
Trends in the weekly incidence of COVID-19 over the first six weeks in four French-speaking countries in West Africa, 2020.

Senegal, Burkina Faso, Guinea, and Mali notified their first cases on March 02, 09, 12, and 25, 2020, respectively. COVID-19 had a different dynamic in the four countries (**Figure 1**). In Guinea, its evolution was marked by an exponential rise in incident cases ranging from 2 (week 1) to 424 (week 6). In Mali, the number of new confirmed cases evolved gradually and linearly; it varied between 28 (week 1) and 188 (week 6). As for Burkina Faso, we observed a polynomial incidence of the pandemic, which increased gradually from 15 cases (week 1) to 147 cases (week 3), then decreased to 118 cases (week 4), increased to 151 cases (week 5) and dropped to 66 cases (week 6). In Senegal, we noted a moderate gradual incidence of the pandemic from 4 cases (week 1) to 80 cases (week 5), which decreased to 58 cases (week 6).

## SCREENING TEST, POSITIVITY RATE AND CASE FATALITY RATE ACROSS THE FOUR COUNTRIES

Over the six weeks, the screening tests performed by Guinea, Senegal, Burkina Faso and Mali were 3923, 2961, 2455 and 2397 respectively. Positivity rates were 25.5%, 23.7%, 22.0% and 9.5% in Mali, Burkina Faso, Guinea and Senegal, respectively. Case fatality rates recorded by Burkina Faso, Mali, Senegal and Guinea were 6.5%, 5.2%, 0.7% and 0.7%, respectively.

## FACTORS THAT MIGHT EXPLAIN THE DIFFERENCES IN THE DISEASE TRAJECTORY ACROSS THE FOUR COUNTRIES

The incidence of COVID-19 in Burkina Faso and Senegal was dropping, while it was increasing slowly in Mali despite the high transmissibility of the virus [6,7], including in asymptomatic or mildly symptomatic carriers [8,9]; however, only in Guinea, it increased rapidly. The trends observed in Burkina Faso, Senegal, and Mali might be due to the early and adequate implementation of containment measures. However, these gains may be reversed if containment measures are lifted without any thorough analysis of the epidemiological situation [10]. This was the case for Senegal, where the curfew was relaxed, restrictions on interurban travel lifted, and certain public places (restaurants, sports halls, movie theaters, etc.) were reopened on June 05, 2020 with 4155 confirmed cases. As a result, the country quickly found itself more exposed than the other three countries with 5090 cases declared positive as of June 14, 2020.

Another reason might be that case detection is not picking up cases anymore. One might posit that at the beginning of the epidemic, the virus spread among the “elites, who had traveled from abroad, or who were in direct contact with people who had traveled from abroad”, and these people were picked up by the epidemiological surveillance system put in place. However, after “4-6” weeks, the virus is being spread through community transmission (far away from the elites), and the existing epidemiological surveillance system is unable to capture those cases.

In contrast, the exponential incidence of COVID-19 in Guinea may be linked to delays in implementing public health response measures [11-13]. While the first confirmed case of COVID-19 was notified on March 12, 2020, the state of health emergency was only declared by the Head of State 18 days later (March 30, 2020) when the country already had 22 confirmed cases and community transmission had already started. This state of health emergency notably enabled the quarantine of Conakry by prohibiting movement towards the countryside, the establishment of a night curfew, and the reduction of the number of passengers by motorbike and by vehicle (taxi, minibus, bus) in order to respect physical distancing. Additionally, shortcomings were noticed, for instance with regard to contact tracing, which could have contributed to the swift and increasing dissemination of the disease in the country. Yet, Guinea should be able to use the lessons learned from the management of the 2014–2016 Ebola outbreak to improve control of the current COVID-19 pandemic. South Korea and Singapore were able to speedily contain COVID-19 by taking advantage of lessons learned from the management of previous outbreaks of Severe Acute Respiratory Syndrome (SARS) and Middle East Respiratory Syndrome (MERS) [14,15]. Guinea could also build on its experience of community engagement in the Ebola response. Indeed, the involvement of community-based organizations and community leaders in response activities contributed to overcoming community reluctance against response actions, a better observance of prevention and control measures, the improvement of contact tracing, and the referral of suspected cases to the Ebola treatment centers [16]. Such an innovative initiative could be adapted to the response to COVID-19, remaining mindful of the differences between Ebola and COVID-19.

**Figure:**
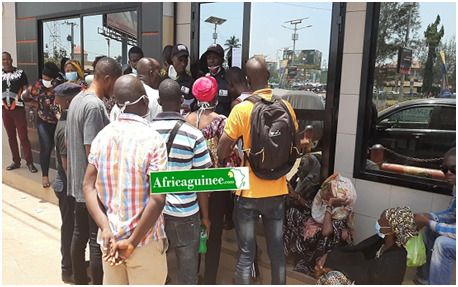
Photo: Customers gathered in front of a bank without respecting physical distancing and not all of them wore face masks, Conakry, Guinea, March 31, 2020 (from Africaguinee.com, used with permission).

Different screening approaches have been used by the four countries. Guinea and Senegal performed more tests compared to Burkina Faso and Mali. This could be explained by the fact that Guinea and Senegal used similar screening strategies based on the detection of suspected cases and identification and testing of their close contacts, increasing, therefore, the number of tests performed. This might result in a lower positivity rate as the denominator increases. In contrast, Mali and Burkina Faso reserved the test only for suspected cases resulting in a higher positivity rate among those tested. The strategy of using large-scale tests has been the cornerstone of response strategies against COVID-19 in countries such as South Korea and Singapore [17]. However, this has been much challenging in West Africa, because of resource constraints, as where the Governments had to rely on local funding. Making the best use of available resources was the guiding principle. Therefore, the four African countries studied gave priority to suspected cases and hospitalized patients which was feasible within their testing capabilities.

Our study reported case fatality rates ranging from 0.7% to 6.5%. These case fatality rates are similar to those existing in the current literature (0.6%-7.2%) [18-20]. In our context, higher case fatality rates have been observed in Mali and Burkina Faso, linked to severe comorbidities and old age, but also due to the lack of adequate supportive treatment, mainly oxygen therapy. Several studies have already mentioned these factors to be associated with high case fatality rates [19,21,22]. The different screening strategies could also explain the variation between the case fatality rates in the four countries studied. When access to screening tests is strictly reserved for suspected cases, the positivity rate is high, and often with advanced disease progression, the case fatality rate among all notified cases is invariably high [19]. Consequently, Burkina Faso and Mali have recorded higher case fatality rates than Guinea and Senegal where screening is also intended for contacts (6.5% and 5.2% in Burkina Faso and Mali, respectively vs 0.7% in Guinea and Senegal).

## CONCLUSION

As the world awaits a potential vaccine to prevent COVID-19, efforts have to focus on slowing the ongoing community transmission through strengthened epidemiological surveillance and reinforcement of infection prevention and control measures. A strategy for mitigation or lifting of the public health response measures is necessary to avoid new peaks or the rebound of the pandemic. Large-scale screening seems relevant to not only facilitate controlling the disease spread but also reducing the case fatality rate by early case detection, prior to the occurrence of complications. It is, therefore, urgent that health authorities in Africa invest in strengthening their health systems in order to better respond to the current pandemic and future health threats.
